# Scan time reduction for readout‐segmented EPI using simultaneous multislice acceleration: Diffusion‐weighted imaging at 3 and 7 Tesla

**DOI:** 10.1002/mrm.25391

**Published:** 2014-07-30

**Authors:** Robert Frost, Peter Jezzard, Gwenaëlle Douaud, Stuart Clare, David A. Porter, Karla L. Miller

**Affiliations:** ^1^ FMRIB Centre, Nuffield Department of Clinical NeurosciencesUniversity of OxfordOxfordUnited Kingdom; ^2^ Siemens AGHealthcare SectorErlangenGermany

**Keywords:** diffusion MRI, readout‐segmented EPI, simultaneous multislice, blipped‐CAIPI, distortion, tractography

## Abstract

**Purpose:**

Readout‐segmented echo‐planar imaging (rs‐EPI) can provide high quality diffusion data because it is less prone to distortion and blurring artifacts than single‐shot echo‐planar imaging (ss‐EPI), particularly at higher resolution and higher field. Readout segmentation allows shorter echo‐spacing and echo train duration, resulting in reduced image distortion and blurring, respectively, in the phase‐encoding direction. However, these benefits come at the expense of longer scan times because the segments are acquired in multiple repetitions times (TRs). This study shortened rs‐EPI scan times by reducing the TR duration with simultaneous multislice acceleration.

**Methods:**

The blipped‐CAIPI method for slice acceleration with reduced g‐factor SNR loss was incorporated into the diffusion‐weighted rs‐EPI sequence. The rs‐ and ss‐EPI sequences were compared at a range of resolutions at both 3 and 7 Tesla in terms of image fidelity and diffusion postprocessing results.

**Results:**

Slice‐accelerated clinically useful trace‐weighted images and tractography results are presented. Tractography analysis showed that the reduced artifacts in rs‐EPI allowed better discrimination of tracts than ss‐EPI.

**Conclusion:**

Slice acceleration reduces rs‐EPI scan times providing a practical alternative to diffusion‐weighted ss‐EPI with reduced distortion and high resolution. Magn Reson Med 74:136–149, 2015. © 2014 The Authors Magnetic Resonance in Medicine published by Wiley Periodicals, Inc. on behalf of International Society for Magnetic Resonance in Medicine.

## INTRODUCTION

Single‐shot echo‐planar imaging (ss‐EPI) is the standard method for acquiring diffusion‐weighted (DW) images 1 due to its low sensitivity to motion artifact and fast imaging capability. The long time between ss‐EPI echoes generates distortions at air–tissue interfaces and the long echo‐trains cause blurring, which limits the echo‐train length and, therefore, the achievable spatial resolution. These effects, which are increasingly problematic at higher field strength, can be mitigated by methods like parallel imaging that reduce the effective echo spacing and echo train length, although the gains achievable in practice with this method are limited by coil geometry. Additionally, the acquisition of k‐space can be segmented to reduce echo‐train length by an arbitrary amount, provided that any velocity dependent phase difference between multiple shots is accounted for with phase navigation 2, 3. Briefly, diffusion‐encoding gradients cause major phase disturbances in the image domain due to tissue deformation during the cardiac cycle 4, 5, 6, 7, which can be measured with a low‐resolution “navigator” image and corrected for in reconstruction 8, 9, 10. Several schemes have been proposed 9, 10, 11, 12, 13, 14, including readout‐segmented EPI (rs‐EPI) 15, which can be modified to acquire a 2D navigator as a second EPI echo train 16, 17. Note that the technique of readout segmentation has also been used to improve the point‐spread function with the GRASE sequence 18. However, these methods all suffer from long scan times because the k‐space acquisition is segmented, thus requiring multiple repetition time (TR) periods to form an image, whereas ss‐EPI collects the entire volume in a single TR. Strategies to accelerate these segmented techniques would thus be very powerful, particularly at 7 Tesla (T), where rs‐EPI has been shown to have increased advantages over ss‐EPI 19.

The rs‐EPI sequence segments the acquisition of k‐space in the readout direction to shorten the echo‐spacing and the echo train duration, thereby reducing phase‐encode distortion artifacts and T_2_* blurring, respectively, in comparison to ss‐EPI. In diffusion tensor imaging (DTI), the number of shots per image limits the number of slices and/or diffusion directions that can be acquired in a reasonable scan time. Readout partial Fourier acquisition has been proposed to accelerate rs‐EPI acquisition times 20, but this incurs an SNR penalty.

A more powerful way to reduce scan time would be to simultaneously excite multiple slices and acquire the combined signal so that fewer slice excitations are required to achieve the same slice coverage 21. This allows direct reduction of the repetition time (TR) by a factor equal to the number of slices simultaneously imaged. This incurs a small reduction in SNR per image due to T_1_ saturation with reduced TR, while SNR efficiency (SNR per square root time) increases substantially up to a maximum at 1.25T_1_ 22. The simultaneous multislice (SMS) method was introduced by Larkman et al. 21 and uses the spatial sensitivity of multichannel array coils 23 to separate the combined slice images with a parallel imaging algorithm. Crucially, SMS acceleration by factor *R* does not incur a √*R* SNR penalty (as occurs in conventional, in‐plane parallel imaging) because k‐space is not undersampled relative to an unaccelerated, single‐slice acquisition. However, the coil (g‐factor) related SNR loss 24 for these slice separation reconstructions can be significant, depending on coil design and slice spacing. For example, it is difficult to achieve high acceleration factors for axial slices in the brain because head coil designs tend to have fewer elements in the superior–inferior direction. SMS using such unfavorable geometries results in simultaneously excited slices with highly similar coil profiles such that voxels cannot be easily separated. The CAIPIRINHA method 25 improves the separability of slice‐aliased voxels by introducing an apparent shift of the simultaneously excited slices relative to each other, such that the aliased locations of two slices experience an effective difference in coil sensitivity due to the in‐plane shift. The apparent shift of slices was initially accomplished in single‐line readouts by altering the RF excitation phase. CAIPIRINHA was subsequently extended to EPI using slice gradients (*G_z_*) during the readout 26. The “blipped‐CAIPI” method 27 was introduced to reduce voxel blurring in the EPI method by using alternating polarity *G_z_* blips to avoid accumulation of phase at the edges of slices during the readout.

Recent studies have used SMS acceleration to increase the number of directions or b‐values for DTI, or to achieve higher temporal resolution for functional MRI 27, 28, 29, 30, 31, 32. In the present work, we demonstrate the use of SMS with blipped‐CAIPI to reduce scan times in rs‐EPI, in which the simultaneous acquisition of multiple slices mitigates the need to acquire each slice with multiple k‐space segments. This technique has the potential to yield high‐resolution images with reduced distortion and blurring without the current burden of long scan times. A preliminary account of this work has been presented recently 33.

This study describes the modifications and considerations required for application of blipped‐CAIPI to the rs‐EPI sequence. Single slice rs‐EPI is compared with SMS rs‐EPI and SMS rs‐EPI image quality and tractography is compared with ss‐EPI. DTI and high‐resolution trace‐weighted data with slice acceleration factor R_slice_ = 2 slice acceleration were acquired at 3T and high‐resolution DTI data with R_slice_ = 3 were acquired at 7T.

## METHODS

The blipped‐CAIPI technique was first described by Setsompop et al. 27. In this section, details of the method relevant to the implementation and reconstruction of blipped‐CAIPI rs‐EPI are discussed, including the changes to the pulse sequence, the reconstruction and the combination with the navigator phase correction. The experiments with the new sequence and the data analysis are then described.

### Blipped‐CAIPI Modifications

**Figure 1:**
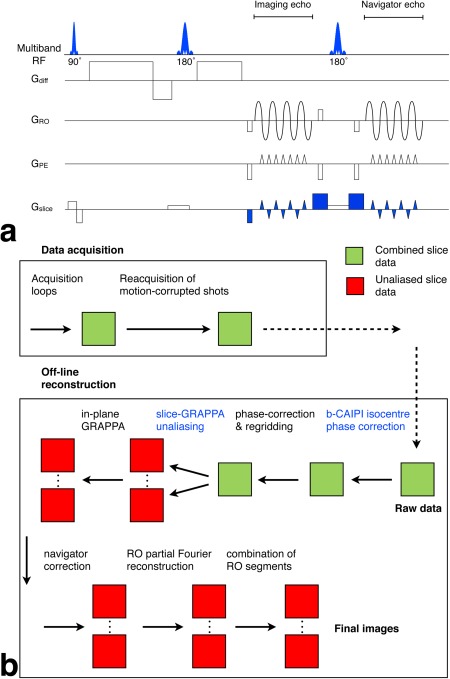
**a**: Blipped‐CAIPI rs‐EPI sequence with modifications to the original rs‐EPI colored blue. These are multiband RF pulses and the slice‐GRAPPA FOV/2 slice‐gradient scheme. **b:** Reconstruction flow chart with blipped‐CAIPI modifications colored blue. Data were acquired as the combined signal (colored green) from multiple slices and the reacquisition was based on these combined navigator data. The combined data were separated into single‐slice data (colored red), with the slice‐GRAPPA unaliasing reconstruction, before the in‐plane GRAPPA reconstruction, navigator correction, POCS reconstruction of partial Fourier acquisitions and combination of readout segments.

The rs‐EPI sequence was modified as shown in Figure 1a to play out multiband RF pulses and blipped‐CAIPI slice gradients. The diffusion preparation module was a modified monopolar scheme 34 to allow a shorter echo time (TE) than a conventional Stejskal‐Tanner preparation 35. Images were reconstructed off‐line in Matlab (Mathworks, Natick, MA) from raw scanner data.

To generate simultaneous multislice excitation and refocusing pulses with a desired slice separation, windowed sinc RF pulses were phase‐modulated and summed. Blipped‐CAIPI modifications were implemented as described in Setsompop et al. 27. An image shift in the phase‐encode direction is introduced to a subset of the excited slices by using the slice gradient (G_z_) to create a slice‐dependent phase variation Δθ between echoes in the EPI echo‐train. G_z_ blips were played out together with the phase‐encode blips (as illustrated in Figure 1a) to impart different amounts of phase at different slice locations. A field of view (FOV)/2 shift was achieved with a π phase difference in the value of Δθ between simultaneously excited slices. The polarity of the blip gradients is alternated so that through‐slice phase variation does not accumulate during the readout and cause blurring. In combination with in‐plane generalized autocalibrating partially parallel acquisition (GRAPPA) acceleration factor R_PE_ = 2, this scheme produces a FOV/2 shift in the 2× undersampled phase‐encode FOV, and hence a FOV/4 shift in the full FOV after reconstruction of missing lines. Slices that are not at G_z_ isocenter have a constant phase offset from the desired 0 – π – 0 ‐ … phase variation between simultaneously excited slices. This phase offset was removed from the raw data at the start of the image reconstruction 27.

The slice‐GRAPPA reconstruction 27 was used to separate the raw slice‐accelerated readout segments and their navigators into single‐slice data. For each of the simultaneously excited slices, separate kernels, or weight sets, were fit in k‐space from the combined data to corresponding reference data acquired without simultaneous excitation. The single‐band reference scans consist of two types of calibration data: phase references for Nyquist ghost correction and calibration data for GRAPPA. The phase reference data consists of a non–phase‐encoded segment for each single‐band slice, while the GRAPPA reference data consists of the central segment for each single‐band slice (note that a single reference data set serves for both slice and in‐plane GRAPPA). Hence, the extra time for reference scans in the SMS acquisition is 2R_slice_ TR periods. In our experiments, these reference acquisition times ranged from 9.2 to 48 s. 3 × 3 kernels were fit using the Nyquist phase‐corrected central segments of the b = 0 data and were then applied to all segments of subsequent b = 0 and diffusion‐weighted volumes of the slice‐accelerated, combined data to reconstruct single‐slice images.

### Multishot Diffusion Corrections

The normal rs‐EPI navigator correction can be performed after separating the readout segments and their navigator echoes into single‐slice data. For reference, the complete data acquisition and reconstruction pipeline is shown in Figure 1b. The basic rs‐EPI reconstruction used was similar to that described in Frost et al. 20, except for the addition of the blipped‐CAIPI phase correction for off‐isocenter slices and slice‐GRAPPA unaliasing. Also, Nyquist ghost correction was performed before regridding, rather than after, which was found to be a slightly more robust correction. For partial Fourier acquisitions with a reduced number of readout segments, POCS reconstruction of the missing data 20 was applied after navigator correction.

In the navigator correction, the segments and navigator echoes are Fourier transformed to image‐space and the phase of the navigator is removed from the corresponding segment's phase. The segments are Fourier transformed back to k‐space so that they can be spliced together to form the full k‐space.

One important feature of our rs‐EPI implementation is the re‐acquisition of segments with excessive motion‐induced phase error, which is assessed in real‐time by quantifying signal spread based on the “distribution width” of the raw navigator data 16. The shots with the worst motion corruption are reacquired 36, 37 to ensure a robust acquisition of artifact‐free DW images. The distribution width, given in Eq. (1), is appealing because it judges motion corruption robustly and it is a fast calculation that can be performed on the raw, undersampled k‐space data in real time without image reconstruction.

(1) $$W_{x}=\int_{i}\int_{k_{y}}\int_{k_{x}}\left|S_{i}(k_{x},k_{y})\right|\cdot \left|k_{x}-\Omega_{x}\right|.$$

In Eq. (1), *S_i_* is the complex k‐space signal in each receive channel *i* and Ω*_x_* is the *k_x_* coordinate of the location with the maximum signal amplitude across all channels. In SMS rs‐EPI, these k‐space data represent multiple aliased slices, which could compromise detection of motion corruption in one of the combined slices, a point that we will return to below.

### Data Acquisition

The diffusion‐weighted SMS rs‐EPI sequence and a ss‐EPI sequence were used to acquire data from two healthy volunteers on a MAGNETOM Verio 3T scanner and another healthy volunteer on an actively shielded MAGNETOM 7T scanner (both from Siemens Healthcare, Erlangen, Germany) under approved technical development ethics protocols. Acquisition parameters of all the sequences used are shown in Table 1, with the overall study design summarized below. In total, SMS rs‐EPI data from seven healthy volunteers and three stroke patients are presented.

**Table 1:** Acquisition Parameters^a^

EPI	Resolution	FOV		Readout	ES	R_slice_ / R_PE_	PE PF	TR / TE	# b=0/ Diff. dir.	Scan time
sequence	(mm^3^)	(cm^3^)	Matrix	segments	(ms)			(s / ms)		(min)
3T protocols										
rs (DTI)	2 iso	22×22×12.6	110×110×58	5	0.32	2 / 2	‐	4.1 / 73	12/60	31
ss	2 iso	19×19×12.6	96×96×58	‐	0.7	‐ / 2	6/8	8.4 / 87	12/60	32 (3 av)
rs	1.5 iso	22×22×9.3	144×144×62	7	0.32	2 / 2	‐	4.8 / 79	12/60	50
ss	1.5 iso	19×19×9.3	128×128×62	‐	0.95	‐ / 2	6/8	13.9 / 88	12/60	54 (3 av)
rs (ADC)	1.7×1.7×5	22×22×13	128×128×26	3	0.40	2 / 2	‐	2.4 / 70	1/3	0:48
rs	1.7×1.7×5	22×22×13	128×128×26	3	0.40	‐ / 2	‐	4.8 / 70	1/3	1:26
rs (patient 1)	1.5 iso	22×22×11.7	146×146×78	5	0.36	2 / 2	‐	9.6 / 60	1/3	4:40
ss	1.8×1.8×2^b^	24×24×13^b^	130×130×50	‐	1.05	‐ / 2	6/8	9 / 98	1/3	2:50 (4 av)
rs (patient 2)	1.5 iso	24×24×11.7	160×160×78	5	0.36	2 / 2	‐	6.9 / 72	1/3	3:20
ss	1.5 iso	24×24×11.7	160×160×78	‐	1.04	‐ / 2	6/8	13.8 / 102	1/3	3:29 (3 av)
rs (TrW)	1×1×5	22×22×13	220×220×26	11	0.32	2 / 2	‐	2.3 / 75	1/3	2:14
ss	1×1×5	22×22×13	220×220×26	‐	1.10	‐ / 2	6/8	7.8 / 80	1/3	0:55
rs (TrW)	1.1×1.1×4	22×22×12	192×192×30	9	0.32	2 / 2	‐	2.7 / 70	1/3	2:20
rs	0.9×0.9×4	22×22×12	256×256×30	11	0.32	2 / 2	‐	3 / 76	1/3	3:05
7T protocols										
rs (DTI)	1.2 iso	19.2×19.2×11.88	160×160×99	6 (6/7 PF)	0.32	3 / 2	‐	8 / 63	4/32	35
ss	1.2 iso	19.2×19.2×11.88	160×160×99	‐	0.82	‐ / 2	6/8	10.8 / 63	12/128	27

a All protocols acquired with b = 1000 s/mm^2^.

b A 0.6 mm gap between slices was used.

#### 3T Experiments

At 3T with R_slice_ = 2, the excitation and refocusing pulse durations were 2.56 ms and 5.12 ms, respectively. A 32‐channel head coil (Siemens Healthcare, Erlangen, Germany) was used for the 3T experiments.

The effect of SMS on rs‐EPI data was evaluated in four subjects by acquiring diffusion‐weighted imaging (DWI) protocols (one b = 0 and three orthogonal b = 1000 s/mm^2^ diffusion directions) with both conventional rs‐EPI and SMS rs‐EPI. Both sequences used 3 readout segments, R_PE_ = 2 in‐plane acceleration and the rs‐EPI pulse durations and TE were matched to the SMS rs‐EPI scans. The resulting ADC maps for conventional and SMS rs‐EPI were compared to investigate any potential image artifacts such as slice leakage or g‐factor noise amplification.

The SMS rs‐EPI sequence was further used to acquire two DTI protocols with 60 isotropically distributed, b = 1000 s/mm^2^ diffusion directions 38 at 2 mm (5 segments) and 1.5 mm (7 segments) isotropic resolution on one subject. Protocols with the same resolution and diffusion directions were also acquired with the ss‐EPI sequence for comparison. Three ss‐EPI averages were acquired to match the scan time of the SMS rs‐EPI. To compare phase‐encode blurring, 1.5 mm isotropic ss‐ and rs‐EPI b = 0 images of a resolution phantom were also acquired.

Finally, we acquired DWI scans in three patients to evaluate clinical feasibility. Different protocol approaches for imaging small infarcts were explored with isotropic resolution compared with high in‐plane resolution and thicker slices. A 1.5 mm isotropic SMS rs‐EPI DWI scan and a 1.8 × 1.8 × 2 mm ss‐EPI scan was acquired in a patient (78 years old) who had suffered a lacunar stroke on the same day. Another patient (71 years old) who had suffered a lacunar stroke on the same day was scanned with matching 1.5 mm isotropic resolution SMS rs‐EPI and ss‐EPI scans. A 1.1 × 1.1 × 4 mm SMS rs‐EPI DWI protocol was acquired in a patient (82 years old) who had suffered a transient ischemic attack (TIA) the previous day. 1 × 1 × 5 mm DWI scans were acquired in one subject with SMS rs‐EPI and ss‐EPI. Two SMS rs‐EPI DWI protocols were acquired with 1.1 × 1.1 × 4 mm and 0.9 × 0.9 × 4 mm in two subjects.

#### 7T Experiments

These experiments were aiming to demonstrate proof of principle at 7T, where T_2_* blurring and distortion necessitate multishot acquisitions for high resolution. However, traditional multiband pulses generated by summing frequency‐modulated pulses could only achieve limited acceleration due to specific absorption rate (SAR) limits. With R_slice_ = 3, the excitation and refocusing pulse durations were increased to 5.12 ms and 10.24 ms, respectively, so that the pulses did not exceed the subject‐specific maximum RF voltage and to reduce the SAR of the pulses. At 7T, acquiring three slices simultaneously did not translate into a 3× acceleration of the scan duration because the TR had to be increased to remain within SAR limits. The scan times were in fact reduced by a factor of 2 with the R_slice_ = 3 acceleration. A 32‐channel head coil (Nova Medical, Wilmington, MA) was used for the 7T experiments.

A comparison between SMS rs‐EPI (six segments with 6/7 readout partial Fourier encoding) and ss‐EPI DTI data was performed at 7T with 1.2 mm isotropic resolution on one subject. At high resolution, scan time becomes particularly problematic for rs‐EPI due to the need for a greater number of readout segments. A reasonable scan time of ∼30 min was targeted, so rs‐EPI at this resolution was limited to 32 directions. Because there is a significant improvement in the quality of diffusion analyses when acquiring more than 30 directions (with more modest gains over 60 directions) 39, we decided to acquire a greater number of directions with ss‐EPI to achieve the fairest comparison. The rs‐EPI and ss‐EPI protocols acquired 32 and 128 directions, respectively, with diffusion‐weighting of b = 1000 s/mm^2^. The number of ss‐EPI directions was chosen such that the ss‐EPI scan time matched the rs‐EPI scan time. The rs‐EPI protocol used 6/7 readout partial Fourier to reduce the scan time per diffusion direction. A b = 0 image of a resolution phantom was also acquired to compare blurring in the rs‐ and ss‐EPI protocols.

### Data Processing and Analysis

The following postprocessing pipeline was applied to the DTI datasets. Eddy current correction was applied to the images using FMRIB's Diffusion Toolbox 40, which consists of an affine registration (12 degrees of freedom) of all volumes to the initial b = 0 s/mm^2^ volume. Nonbrain tissue was removed from the images using Brain Extraction Tool 41, and the diffusion tensor was fitted to the reconstructed data using FMRIB's Diffusion Toolbox. Fractional anisotropy (FA) maps were calculated and for tractography BEDPOSTX 42 was used to generate probability density distributions on two crossing fiber orientations at each voxel. In the 3T data, tractography was seeded in the subject's structural space for several major association, commissural and projection fibers: the superior longitudinal fasciculus (SLF), cingulum bundle, optic tract, fornix, corpus callosum (CC), and corticospinal tract (CST). Also, SNR and blurring were quantified in the raw images from the DTI protocol. SNR was estimated from the b = 0 s/mm^2^ images that were interleaved with the diffusion scans (the number of b =0 s/mm^2^ images in each protocol is given in Table 1). Voxel‐wise SNR was estimated by dividing the mean signal across b = 0 s/mm^2^ repeats by the standard deviation in signal. The mean and standard deviation within the brain extracted mask were taken as the SNR estimate and the error, respectively. Resolution was estimated using the *smoothest* tool in the FMRIB Software Library (http://www.fmrib.ox.ac.uk/analysis/techrep/tr00df1/tr00df1/node4.html). The mean pixel size across all b = 0 s/mm^2^ images was estimated with *smoothest* and normalized to the rs‐EPI estimate within each resolution. For comparison with experimental measurements, echo‐train parameters were used to calculate the theoretically expected percentage of voxel blurring and the effective phase‐encode resolution, Δy′, using the following formula for spin‐echo EPI 43:

(2) $$\Delta y^{\prime }=\Delta y\left(\frac{\sqrt{3}}{T_{2}}+\frac{1}{T_{2}^{\prime }}\right)\frac{T_{acq}}{\pi }$$

where Δy is the nominal voxel size, *T_acq_* is the duration of the readout and *T*_2_ and *T*_2_′ are the decay constants of the thermodynamic and external field induced effects, respectively. The total loss of resolution is approximated as the result of two separate *T*_2_ and *T*_2_′ filters. Based on *T*_2_* = 47 ms and *T*_2_ = 80 ms for 3T 44 and *T*_2_* = 28 ms 45 and *T*_2_ = 48.5 ms 46 for 7T, white matter *T*_2_′ values of 114 ms at 3T and 66 ms at 7T were calculated according to:

(3) $$\frac{1}{T_{2}^{*}}=\frac{1}{T_{2}}+\frac{1}{T_{2}^{\prime }}.$$

For the DWI data, trace‐weighted images were calculated by taking the geometric mean of the three b = 1000 s/mm^2^ images acquired with orthogonal diffusion weightings. Maps of apparent diffusion coefficient (ADC) were generated from the b=0 (S_b0_) and trace‐weighted images (S_trw_) as follows:

(4) $$ADC=-\frac{\ln(S_{trw}/S_{b0})}{1000}.$$

## RESULTS

### Basic Implementation of SMS rs‐EPI

**Figure 2:**
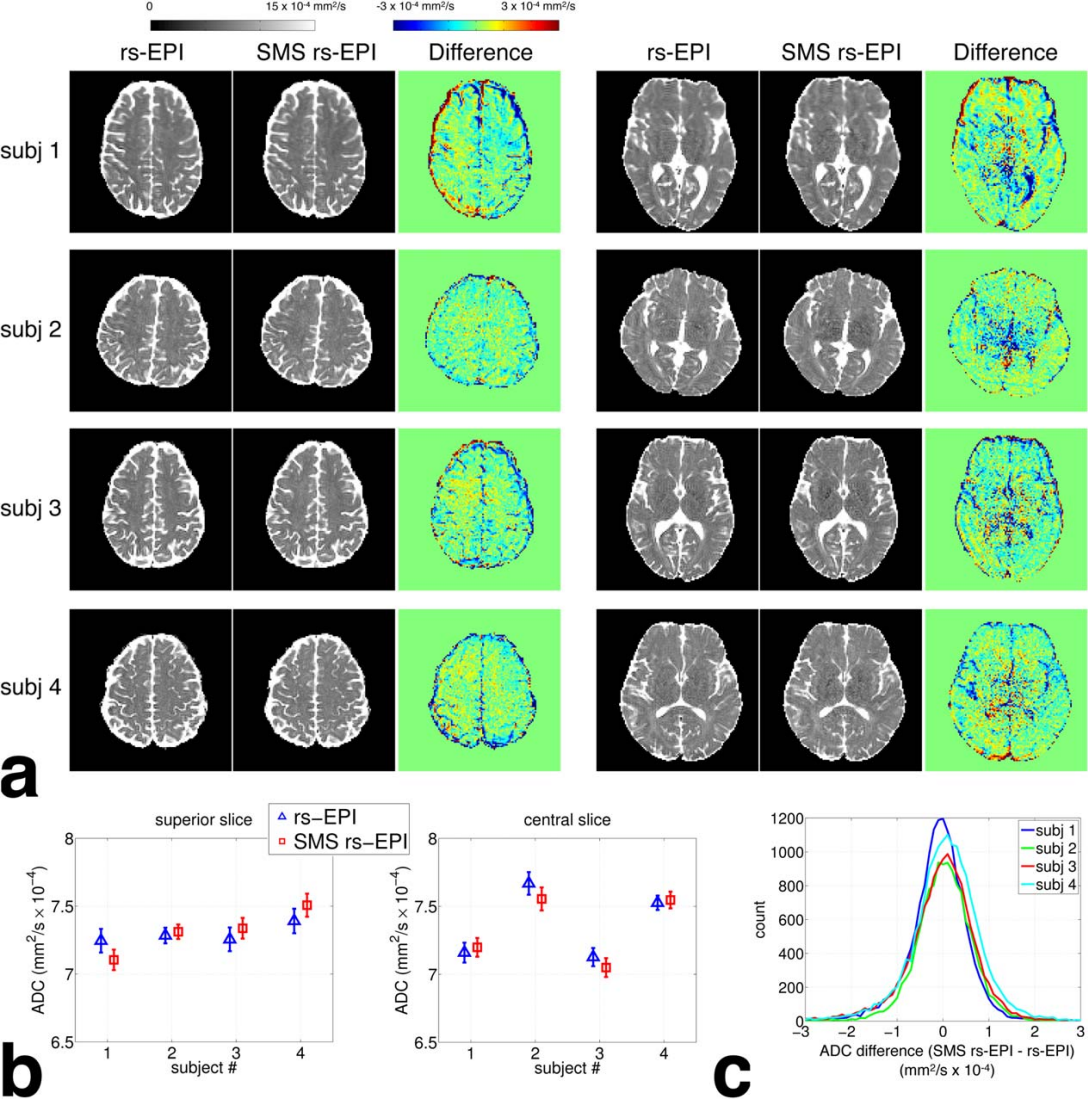
Comparison of ADC maps between rs‐EPI and SMS rs‐EPI. **a:** ADC maps and the difference between rs‐EPI and SMS rs‐EPI data. **b:** Plots of mean ADC in white matter regions of interest (ROI) in the slice positions shown in panel a). The error bars show the confidence interval (*P* < 0.05) on the estimate of the mean. **c:** Histograms of the ADC difference between SMS rs‐EPI and rs‐EPI in white matter ROIs in all slices.

A comparison of ADC between rs‐EPI and SMS rs‐EPI in four subjects is shown in Figure 2. Visual inspection of the images in Figure 2a suggests that the SMS rs‐EPI data are very similar to the rs‐EPI data and that the SMS acceleration does not introduce serious artifacts. The mean ADC values in white matter for the slices shown in part a) are plotted in Figure 2b. Histograms of the ADC difference in white matter in all slices between SMS rs‐EPI and rs‐EPI are shown in Figure 2c. These plots show that the ADC estimates are well matched in rs‐EPI and SMS rs‐EPI when the scan time (including slice unaliasing, GRAPPA and Nyquist correction calibration TRs) is reduced from 1:26 min to 48 s with slice acceleration.

**Figure 3:**
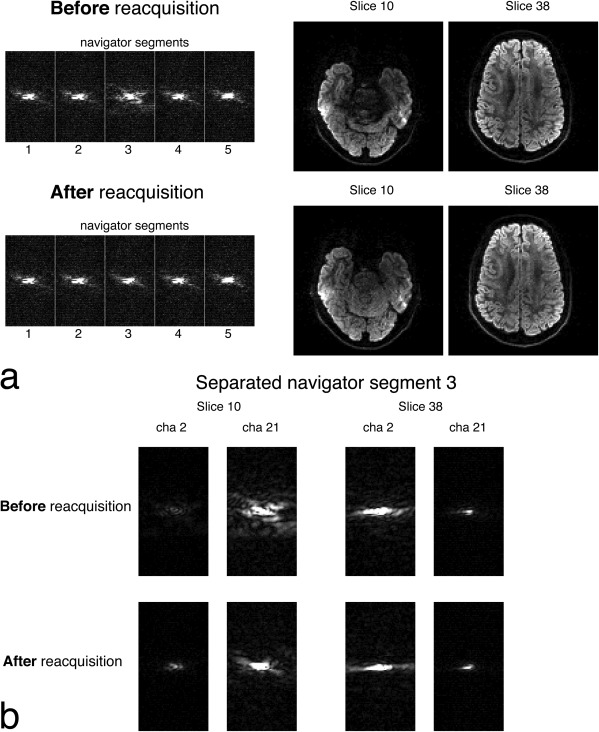
Navigator‐based reacquisition with two simultaneously excited slices (labelled with their anatomical slice number) with b = 1000 s/mm^2^ diffusion‐weighting. **a:** Before reacquisition, corruption in segment 3 is indicated by the dispersed k‐space in the navigator, which contains combined data from the two slices. When segment 3 is reacquired, artifacts are removed from the images. **b:** Single‐channel maps (all at the same scale) of the unaliased k‐space from navigator segment 3. Two channels are chosen which are close to slice 10 (channel 21) and 38 (channel 2) to demonstrate the variation in signal.

As mentioned above, one aspect of our rs‐EPI approach that could be compromised by SMS excitation is the reacquisition scheme, in which corrupted data are identified based on navigator distribution width and reacquired subsequently. In SMS rs‐EPI, we chose not to separate navigators for different slices to enable rapid computation of the distribution width. For the relatively small number of simultaneously excited slices tested here, the distribution width was found to be very successful at identifying motion‐corrupted data for reacquisition (as judged by visually inspecting all images). An example of the detection of motion corruption with R_slice_ = 2 acceleration during the on‐line reacquisition is shown in Figure 3. In Figure 3a, before reacquisition, the navigator for readout‐segment number 3 (the central readout segment) shows more dispersed k‐space signal than the other navigators. Based on the distribution width metric, this segment was judged to be one of the 20% most corrupted of all the segments so it was marked for reacquisition. When this segment was reacquired, there was less motion corruption (indicated by the reduced navigator spread) and the signal dropout in the center of slice 10 is restored. This example, which was acquired with diffusion encoding close to the superior–inferior axis of the brain, highlights the nature of motion‐phase artifacts in diffusion, which are mainly due to the pulsatile deformation of the brain during systole 5, 6, 7, 10. It also shows the importance of having cleanly acquired data close to the center of k‐space to minimize artifacts and for this reason the navigator reacquisition is preferentially weighted to central segments. The final (postreacquisition) diffusion‐weighted images were visually inspected for artifacts (like Figure 3a) due to possible failure of the reacquisition. No such artifacts were observed in any of the images, indicating that the straightforward analysis of the aliased k‐space data spread is able to detect motion corruption at these low acceleration factors, even if there is only significant motion in one of the excited slices within the composite multislice dataset.

In Figure 3b, we examine this specific case in more detail. The segment marked for reacquisition (navigator segment number 3 from Figure 3a) has been unaliased and the signal is shown from coil elements 2 and 21, which are close to slices 38 and 10, respectively. In the unaliased slice 10 navigators, the proximity of the slice to the coil element results in high signal in channel 21 and a clear depiction of the dispersion of k‐space due to motion. These unaliased navigators are included to demonstrate that the detection of motion corruption could be performed separately on each of the simultaneously excited slices to improve the sensitivity to slice‐specific effects, although this would require a more sophisticated reconstruction. With higher slice acceleration factors, identification of motion corruption may only be possible in unaliased slice data or with coil‐specific k‐space distribution widths to consider the signal (and hence corruption) from more localized regions of the coil.

### Comparison of SMS rs‐EPI and ss‐EPI

**Figure 4:**
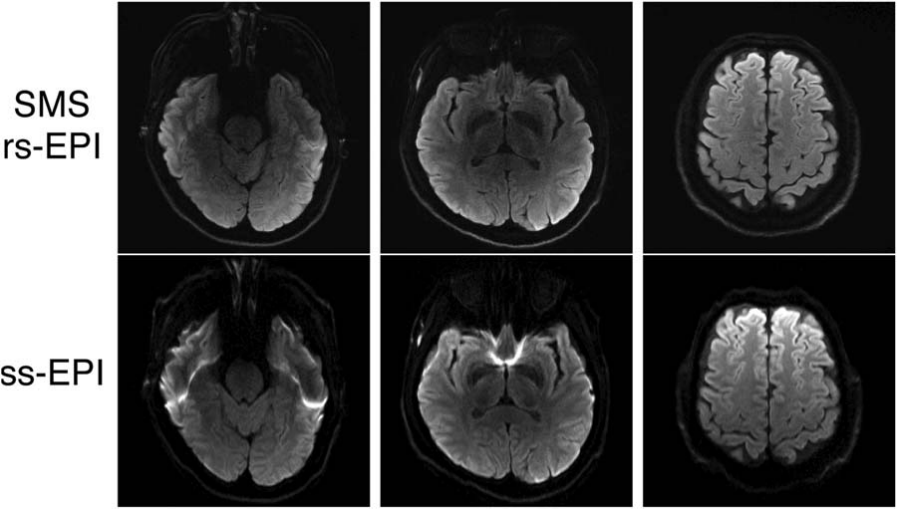
Comparison of 1 × 1 × 5 mm trace‐weighted images acquired with SMS rs‐EPI and ss‐EPI. Improvements with SMS rs‐EPI in typical distortion regions are shown in left and center columns. A more superior slice is shown in the right column where there are less ss‐EPI distortions but the SMS rs‐EPI images show improved sharpness.

Figure 4 compares 1 × 1 × 5 mm trace‐weighted images acquired with SMS rs‐EPI and ss‐EPI. The left and center columns show the typical distortion and signal pile‐up regions in ss‐EPI and the improvement with SMS rs‐EPI. The right‐hand column demonstrates that ss‐EPI distortions are reduced in more superior regions, however, the impressive sharpness of the SMS rs‐EPI data is evident. Note that the ss‐EPI images (here and in all ss‐EPI images presented) are reconstructed with zero‐filled partial Fourier reconstruction. This is a standard approach in commercial applications of DW ss‐EPI to avoid possible artifacts due to motion‐induced phase error in partial Fourier reconstruction algorithms.

**Figure 5:**
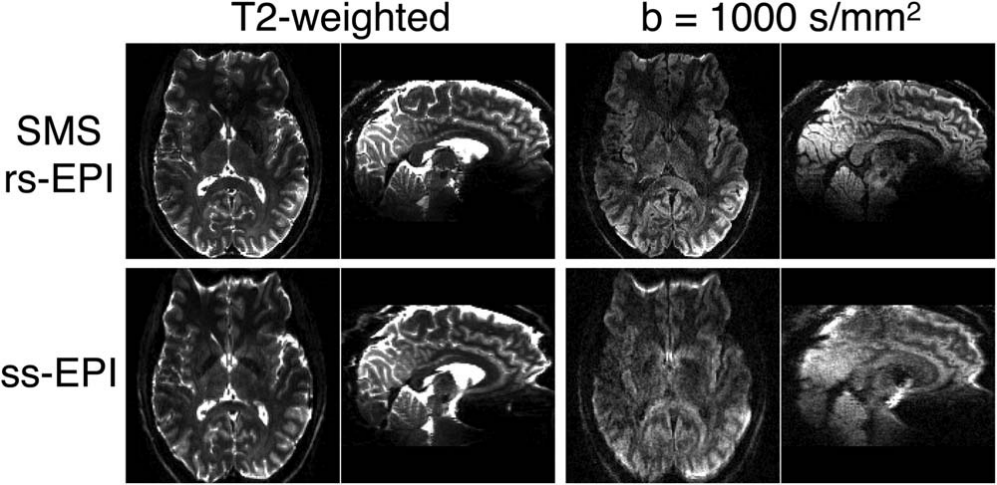
Examples of raw T_2_ weighted and b = 1000 s/mm^2^ images acquired at 7T with the ss‐EPI and SMS rs‐EPI DTI protocols with 1.2 mm isotropic resolution.

Raw images acquired with the 3T DTI protocols are shown in the online Supporting Figure S1, which is available online. These demonstrate the reduced blurring and distortion in the rs‐EPI data due to the shorter echo train duration and echo‐spacing, respectively, but also the higher SNR that is achieved with three ss‐EPI averages in the same scan time. Raw images acquired at 7T are shown in Figure 5 demonstrating that the improvement in rs‐EPI image quality relative to ss‐EPI increases at higher field. The increased blurring in ss‐EPI is quantified in Supporting Table S1, which presents a comparison of SNR and resolution in the raw b = 0 s/mm^2^ images. The estimated ss‐EPI phase‐encode pixel sizes are larger than rs‐EPI by 27% at 2 mm resolution, 46% at 1.5 mm resolution, and 50% at 7T 1.2 mm resolution. Based on the echo‐train durations in the presence of T_2_ and T_2_* decay, the nominally 1.5 mm ss‐EPI pixels are expected to be blurred by 44% compared with 22% blurring for rs‐EPI. For the nominally 1.2 mm voxels at 7T, this increases to 79% blurring for ss‐EPI and 41% for rs‐EPI. Some SNR variation between ss‐ and rs‐EPI (see Supporting Table S1) is expected to reflect the different amounts of blurring (with SNR increasing linearly with the breadth of the blurring kernel in the phase‐encode direction). The blurring in the ss‐EPI images is a combination of T_2_* effects and smoothing due to the zero‐filled phase‐encode partial Fourier reconstruction. A further comparison of phase‐encode blurring at 1.5 mm (3T) and 1.2 mm (7T) nominal resolutions is shown in Supporting Figure S2. The same shim settings were used for the ss‐ and rs‐EPI acquisitions. The increased blurring and distortion in ss‐EPI is evident in the images and the profiles of the signal through the red dotted lines highlight the ability of rs‐EPI to resolve small structures. Signal that is represented by one voxel in the rs‐EPI images is blurred into the neighboring two voxels in the ss‐EPI images.

### Trace‐Weighted SMS rs‐EPI of Vascular Pathology

**Figure 6:**
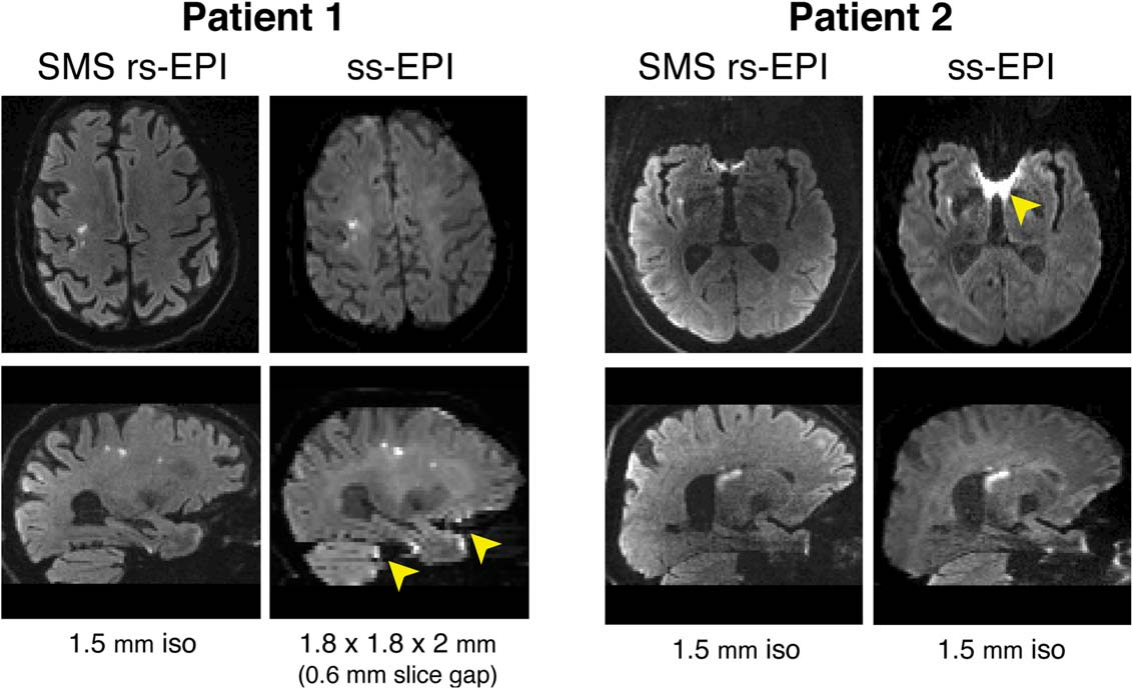
Comparison of SMS rs‐EPI and ss‐EPI and in two lacunar stroke patients. Regions in ss‐EPI images where distortion and signal pile‐up is worse than SMS rs‐EPI (indicated by yellow arrows) appear similar to stroke lesions.

A comparison of stroke imaging with SMS rs‐EPI and ss‐EPI is presented in Figure 6. In scans on patient 1, a 1.5 mm isotropic SMS rs‐EPI acquisition is compared with a 1.8 × 1.8 × 2 mm ss‐EPI scan that was part of a longitudinal clinical study of lacunar stroke. Matched 1.5 mm isotropic resolution scans were compared on the second patient. The similarity between true stroke lesions and distortion‐induced signal pile‐up suggests that rs‐EPI could be particularly useful in the lower brain and neck regions. Supporting Figure S3 demonstrates the potential of SMS rs‐EPI for further improvements in the resolution of trace‐weighed images in two healthy subjects and one TIA patient.

### High Angular Resolution SMS rs‐EPI of White Matter

For the major association, commissural and projection fibers, probabilistic tractography generally improved at 1.5 mm compared with 2 mm in both the rs‐ and ss‐EPI, producing more true positive tracks and fewer false positive tracks. For example, in 1.5 mm rs‐ and ss‐EPI data, tractography seeded in the anterior pillars of the fornix correctly tracked into the hippocampus and did not mistrack into the anterior commissure, but not in 2 mm data.

**Figure 7:**
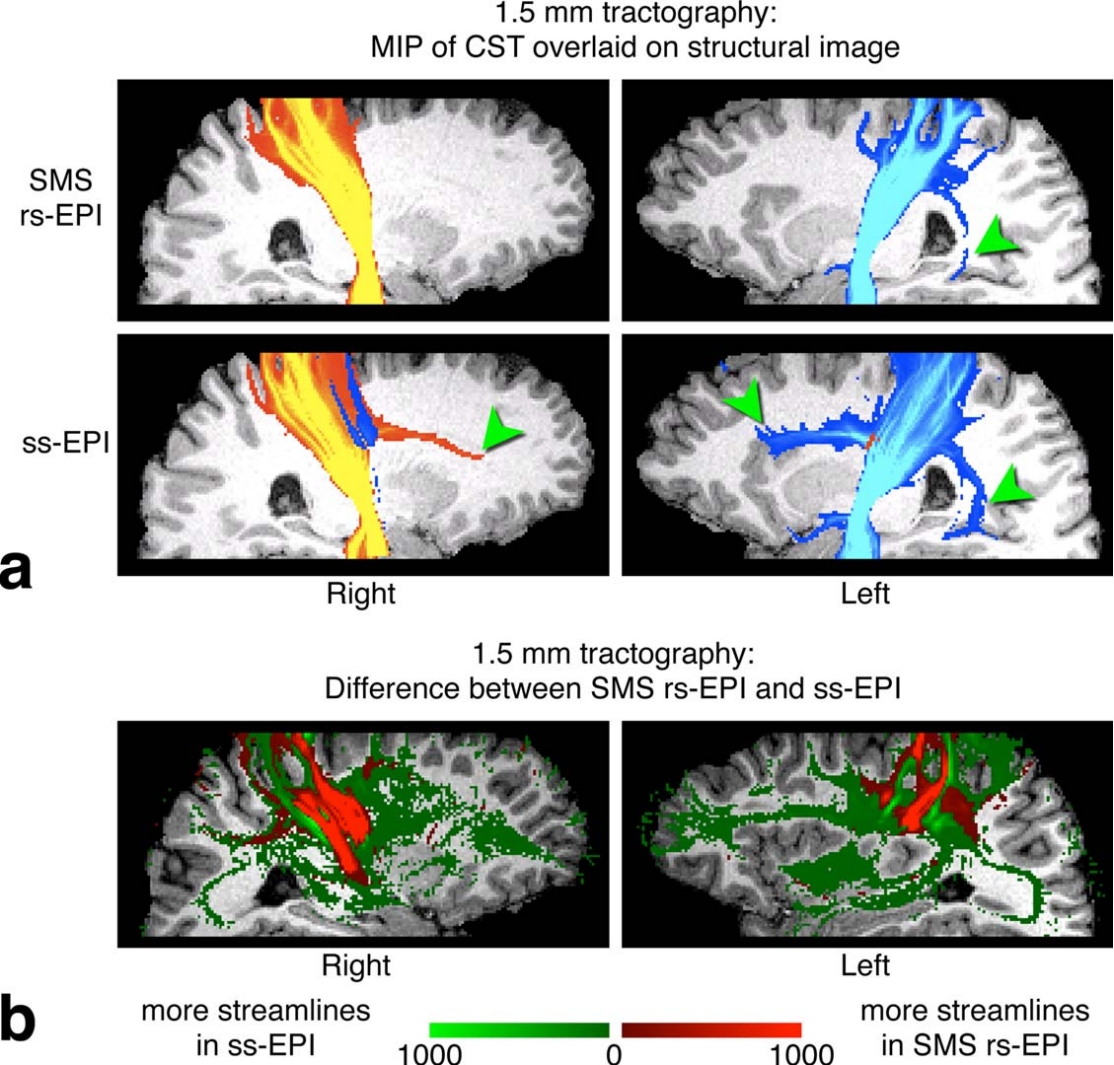
3T results from the 1.5 mm isotropic ss‐ and rs‐EPI acquisitions. **a:** Tractography comparison in the CST. Maximum intensity projections (within‐hemisphere) of the CST are shown overlaid onto representative slices of the structural image. Streamlines seeded in the left and right hemispheres are shown in blue–light blue and red–yellow, respectively, at the same threshold (>100 streamlines). Green arrows indicate mis‐tracking into the SLF, in regions where both CST and SLF cross. **b:** Difference between rs‐ and ss‐EPI tractography in the CST in a representative slice. Regions where there are more rs‐EPI streamlines are shown in red. Regions where there are more ss‐EPI streamlines are shown in green.

Tractography in the 1.5 mm rs‐ and ss‐EPI datasets was comparable in terms of the ratio of true to false positives. The streamlines generally died out earlier in the rs‐EPI data, such that the total number of streamlines reaching cortex (whether true or false positives) was greater in ss‐EPI. This effect is thought to be attributable to the lower SNR in the rs‐EPI data. On the other hand, we found that dispersion of tracts was higher in the ss‐EPI data. For instance, rs‐EPI yielded improved tractography in the CST (Fig. 7). In regions where CST and SLF cross (centrum semiovale), the CST mistracked anteriorly into the SLF in ss‐EPI (Figure 7a, arrows), while the CST projections into M1 and S1 cortex were much more cleanly identified with rs‐EPI. The difference between rs‐EPI and ss‐EPI tractography of the left and right CST in representative slices is shown in Figure 7b. In regions that are plausible segmentations of CST, rs‐EPI tracts “dispersed” less than ss‐EPI, so that generally and in the example of Figure 7b, in the center of tracts, there were more rs‐EPI streamlines, and at the periphery there were more ss‐EPI streamlines. This could be a consequence of the lower blurring in rs‐EPI. Although the number of tracts reaching all regions of cortex was higher in ss‐EPI, the greater mistracking was to a degree such that the fidelity of the tract reconstruction was overall superior in rs‐EPI.

**Figure 8:**
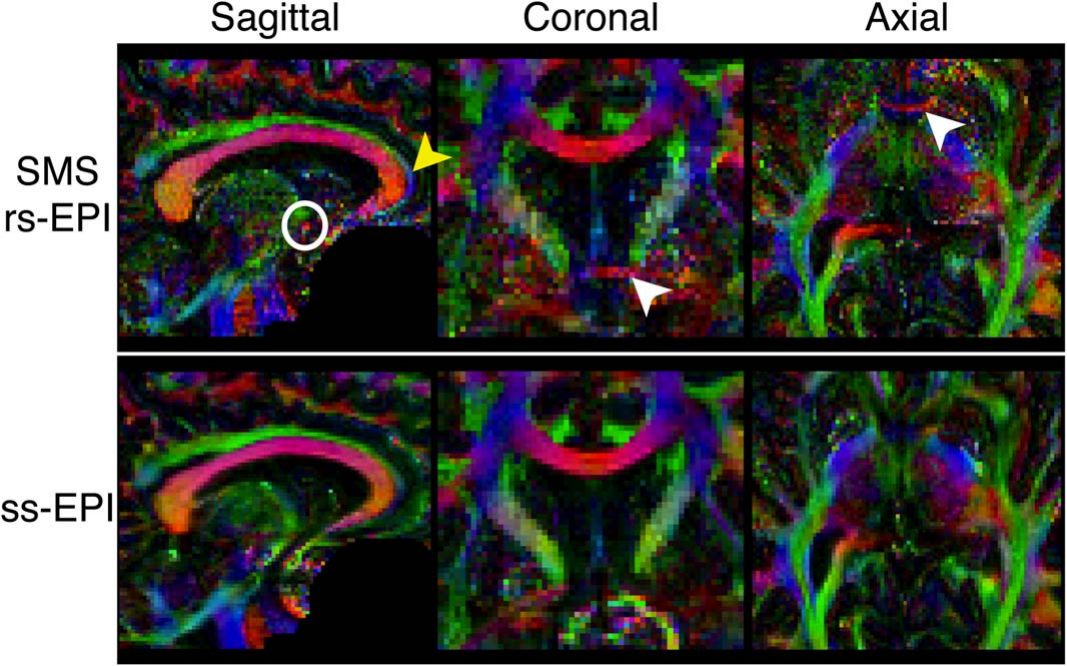
7T DTI results comparing blurring and distortion in the 1.2 mm isotropic ss‐ and rs‐EPI acquisitions. The rs‐EPI data show the anterior commissure (white circle and arrows), which is not present in the ss‐EPI data. rs‐EPI also exhibits reduced blurring and distortion in the cingulum (yellow arrow). Color‐coded maps of the principal eigenvector are shown. Color code: green, anterior–posterior; blue, superior–inferior; red, left–right.

**Figure 9:**
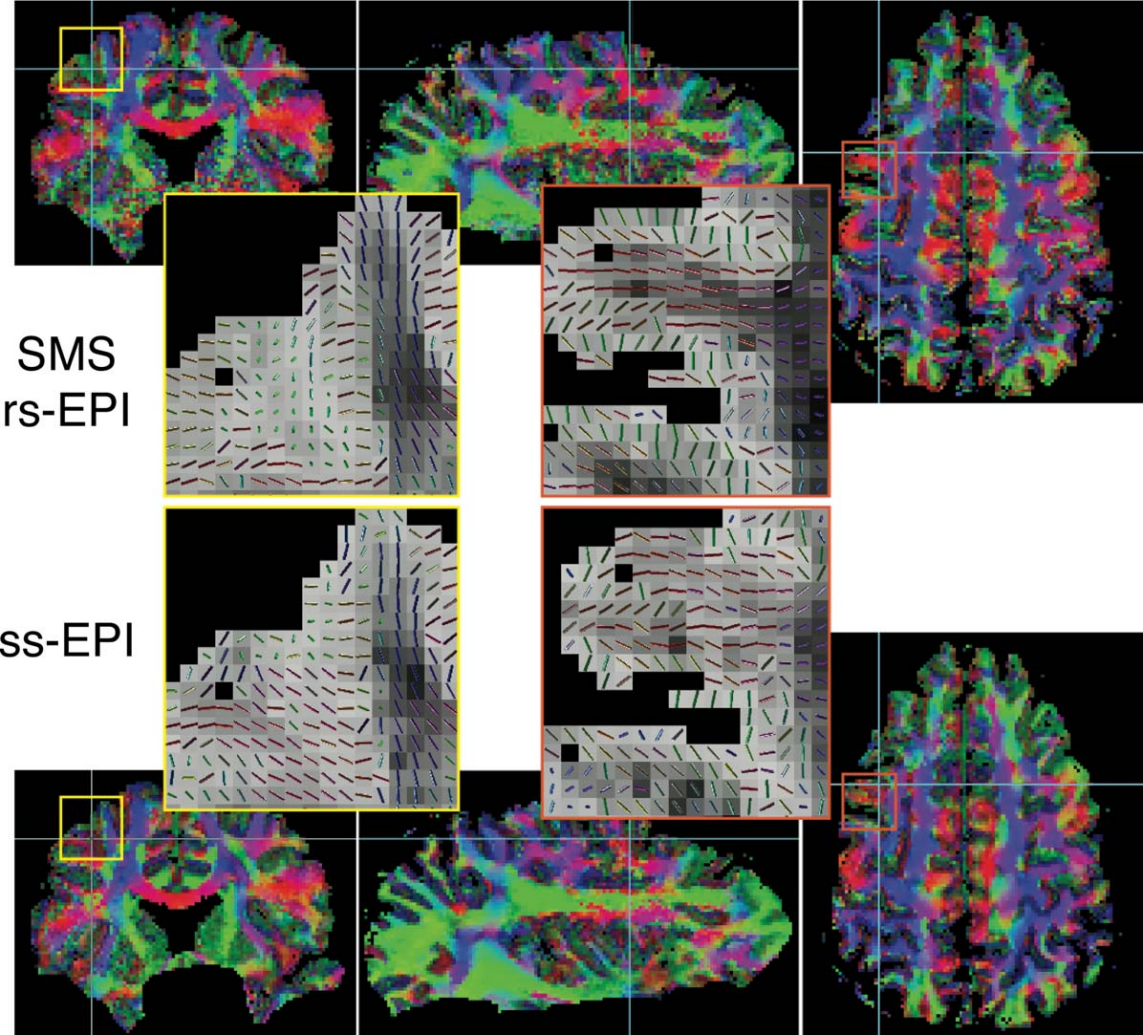
7T DTI results comparing cortical anisotropy in the 1.2 mm isotropic ss‐ and rs‐EPI acquisitions. The rs‐EPI data highlight the radial cortical anisotropy with vectors turning as they enter the cortex to be perpendicular to the cortical surface. Color‐coded maps of the principal eigenvector are shown as well as zoomed regions of vectors overlaid on fractional anisotropy (FA) maps. Note that the FA maps in the zoomed regions are inverted to show the cortex as bright. Color code: green, anterior–posterior; blue, superior–inferior; red, left–right.

An example of the benefits of reduced blurring and distortion in rs‐EPI at 7T is shown in the 7T DTI results in Figure 8. The rs‐EPI data resolves the thin anterior commissure between hemispheres whereas the tract is not visible in the ss‐EPI data. The Figure also demonstrates the reduced blurring and distortion in tracts such as the cingulum. Figure 9 shows that rs‐EPI has a closer correspondence to the expected fiber structure at the cortex boundary. rs‐EPI vectors are more homogeneous in fiber tracts and are perpendicular to the cortical surface as they enter the banks of the sulci, whereas the ss‐EPI vectors are in some cases tangential to the cortical surface.

## DISCUSSION

This study demonstrates that a blipped‐CAIPI SMS modification is compatible with the rs‐EPI sequence and, in particular, with multishot navigator corrections and navigator‐based reacquisition. Comparison of ADC maps in Figure 2 showed that SMS acceleration does not introduce serious artifacts and the data are well matched to single‐slice rs‐EPI with respect to image quality. SMS has previously been used to accelerate diffusion‐weighted ss‐EPI acquisitions and it is now expected to address one of the major current shortcomings of rs‐EPI: long scan times, which can be prohibitive. Acquisition of whole‐brain, high‐resolution, trace‐weighted data in clinically relevant scan times is now possible, as are measurements with a large number of diffusion directions. DWI results in Figures 4 and 6 suggest that faster DWI scans with reduced distortion and signal pile‐up could be useful for clinical imaging in regions of field inhomogeneity, such as frontotemporal cortex, brainstem and spine. We have also demonstrated the feasibility of tractography from high‐resolution rs‐EPI data. Although ss‐EPI streamlines had a greater tendency to reach cortex, likely as a consequence of higher SNR, the rs‐EPI streamlines were more faithful to the local anatomy, as seen in Figure 7. At 3T, differences between rs‐EPI and ss‐EPI were subtle for many tracts, but higher resolution in all cases improved the accuracy of tractography. It is thus significant that rs‐EPI does not suffer from the distortion‐resolution tradeoff that limits ss‐EPI. The 7T DTI results show clear benefits of the resolution and quality of the rs‐EPI data, although the SNR is noticeably higher in the ss‐EPI diffusion parameters. For example, it was possible to resolve the anterior commissure in rs‐EPI data, but not in ss‐EPI data, which is consistent with the findings of a previous 7T rs‐EPI study 19. The high ss‐EPI SNR is due to the higher number of directions achievable in the same scan time (128 compared with 32) and is also expected to be increased by blurring caused by T_2_* decay and zero‐filled phase‐encode partial Fourier reconstruction. Overcoming SAR limitations, as discussed below, to reduce TR will improve the SNR per unit time of the 7T rs‐EPI DTI.

Combining across ss‐EPI images with reversed phase‐encode directions would enable some distortion reduction 47. However, these correction algorithms are unable to remove extreme distortions in which the local “true” field Jacobian approaches zero or becomes negative (corresponding to signal pile‐up or a reversal of the order of signal in the image, respectively), which can occur in high‐resolution or high‐field acquisitions. It is therefore preferable to minimize artifacts at the acquisition stage. Our tractography results further suggest that there are benefits of the reduced blurring in rs‐EPI data.

High resolution does still pose an SNR challenge, which motivates higher SMS acceleration factors to increase SNR efficiency. SMS acceleration at high field poses additional challenges. Higher slice acceleration will require RF pulse designs to address limits on SAR and peak B_1_, especially at higher field because SAR scales with B_0_^2^ and B_1_^2^. RF pulses could be designed with VERSE 48 to reduce the peak RF voltage and SAR, however, this adversely affects the slice profile, particularly at higher field where there is increased B_0_ inhomogeneity. Lengthening the pulses reduces SAR but also increases sensitivity to off‐resonance effects in the excited slices. For the simple summation of phase‐modulated pulses used in this study, the peak RF voltage (which scales linearly with the number of slices) and SAR were usually close to the scanner‐imposed limits (which are calculated on a subject‐specific basis).

The amount of slice acceleration used will depend on TR limitations and what is deemed an acceptable level of slice separation artifact 22. As mentioned above, the SNR efficiency peaks at a TR value of approximately 1.25T1, so acceleration factors that achieve this TR are sufficient. For whole‐brain imaging, thick‐slice trace weighted acquisitions with low numbers of slices 25, 26, 27, 28, 29, 30 can use slice acceleration factors of 2–3 to operate in this TR regime of 1–2 s at 3T. Thin‐slice DTI acquisitions, which require more slices and hence longer TR (∼10 s), would be candidates for higher acceleration factors. Increasing slice acceleration and/or combining with in‐plane parallel imaging, will lead to more g‐factor noise amplification and residual slice aliasing, as discussed below. Protocols that use in‐plane parallel imaging to minimize TE and susceptibility artifacts will therefore have tighter constraints on slice acceleration.

The success of the slice separation reconstruction relies primarily on the geometry of the receive coil used, in particular the spatial sensitivity variation between elements in the slice direction and, when blipped‐CAIPI is used, in‐plane. The simultaneous use of in‐plane parallel imaging with blipped‐CAIPI will in general present a tradeoff between the two types of acceleration. Blipped‐CAIPI spreads the slice‐aliasing pattern in the phase‐encode direction to create additional sensitivity variation between aliased voxels, thereby reducing the g‐factor in the unaliasing reconstruction. There is interaction between the aliasing patterns due to in‐plane and slice acceleration and less scope to spread the slice‐aliasing in the reduced phase‐encode field of view, resulting in increased residual artifacts and g‐factor 22. However, the SNR performance of SMS acquisitions (un‐shifted and blipped‐CAIPI) has been shown to improve at higher field strength 27, and with coil designs optimized for parallel imaging 49, where in both cases the improvement is due to increased spatial orthogonality between coil elements.

At 7T, it was necessary to increase TR to comply with SAR limits, such that our three‐slice simultaneous excitation in practice only resulted in a factor of two reduction in scan time. The recently introduced power independent of number of slices (PINS) pulses 50 have SAR that is approximately independent of the number of simultaneously excited slices. An RF pulse envelope is multiplied with a comb function (the result is a sampled pulse envelope) so that a series of repeating slices is excited within the range of the transmit coil. PINS pulses have the disadvantages of limited bandwidth (due to the blipped gradient waveforms) and restrictions on slice geometry (which can be overcome if PINS pulses are only used for refocusing 51). As a consequence of the low bandwidth, we expect there to be significant challenges when using these pulses to excite thin slices (for high‐resolution isotropic DTI) in the presence of 7T off‐resonance. We plan to explore the use of PINS with rs‐EPI in future work. Simultaneous multislice excitation using parallel transmission has also been demonstrated recently as a strategy for reducing RF power and/or improving B1+ homogeneity 52, 53.

In the rs‐EPI approach, minor motion corruption (outside of systole) is removed by the nonlinear navigator correction, while segments with more significant corruption (during systole) are simply reacquired. The navigator‐based reacquisition was able to identify the shots with the most motion corruption based on the aliased navigator signals from the simultaneously acquired slices. Lower slices in the brain suffer the worst motion artifact 5, 6, 7, 10, so as the number of simultaneously acquired slices is increased it may be necessary to modify the real‐time detection of motion corruption to robustly detect dropout in a subset of the slices. With higher acceleration factors, the presence of just one corrupted slice causes that corruption to be a smaller fraction of the total signal making it harder to detect. In such cases, the signal from individual receive channels could be considered in a more detailed way based on their proximity to the slices, rather than summing all coil contributions to calculate one distribution width. Alternatively, the data could be unaliased in real‐time and the distribution widths calculated for the k‐space signal from each slice.

## CONCLUSIONS

In this study, we have applied the blipped‐CAIPI SMS method to rs‐EPI to realize 50% reduction in scan time with potential for further acceleration. We have shown slice acceleration to be compatible with the multishot diffusion approach, and the shorter scans mean that using SMS rs‐EPI for high‐resolution trace‐weighted imaging and tractography is now a practical alternative to ss‐EPI, offering reduced distortion and blurring. Further work on achieving higher slice acceleration factors will improve the SNR per square root time.

## Supporting information

Supplementary Information

## References

[1] Turner R , Le Bihan D , Chesnick AS . Echo‐planar imaging of diffusion and perfusion. Magn Reson Med 1991;19:247–253. 1881311 10.1002/mrm.1910190210

[2] Ordidge RJ , Helpern JA , Qing ZX , Knight RA , Nagesh V . Correction of motional artifacts in diffusion‐weighted MR images using navigator echoes. Magn Reson Imaging 1994;12:455–460. 8007775 10.1016/0730-725x(94)92539-9

[3] Anderson AW , Gore JC . Analysis and correction of motion artifacts in diffusion weighted imaging. Magn Reson Med 1994;32:379–387. 7984070 10.1002/mrm.1910320313

[4] Feinberg DA , Mark AS . Human brain motion and cerebrospinal fluid circulation demonstrated with MR velocity imaging. Radiology 1987;163:793–799. 3575734 10.1148/radiology.163.3.3575734

[5] Greitz D , Wirestam R , Franck A , Nordell B , Thomsen C , Stahlberg F . Pulsatile brain movement and associated hydrodynamics studied by magnetic resonance phase imaging. The Monro‐Kellie doctrine revisited. Neuroradiology 1992;34:370–380. 1407513 10.1007/BF00596493

[6] Enzmann DR , Pelc NJ . Brain motion: measurement with phase‐contrast MR imaging. Radiology 1992;185:653–660. 1438741 10.1148/radiology.185.3.1438741

[7] Poncelet BP , Wedeen VJ , Weisskoff RM , Cohen MS . Brain parenchyma motion: measurement with cine echo‐planar MR imaging. Radiology 1992;185:645–651. 1438740 10.1148/radiology.185.3.1438740

[8] Butts K , Pauly J , de Crespigny A , Moseley M . Isotropic diffusion‐weighted and spiral‐navigated interleaved EPI for routine imaging of acute stroke. Magn Reson Med 1997;38:741–749. 9358448 10.1002/mrm.1910380510

[9] Pipe JG , Farthing VG , Forbes KP . Multishot diffusion‐weighted FSE using PROPELLER MRI. Magn Reson Med 2002;47:42–52. 11754441 10.1002/mrm.10014

[10] Miller KL , Pauly JM . Nonlinear phase correction for navigated diffusion imaging. Magn Reson Med 2003;50:343–353. 12876711 10.1002/mrm.10531

[11] Liu C , Bammer R , Kim D , Moseley ME . Self‐navigated interleaved spiral (SNAILS): application to high‐resolution diffusion tensor imaging. Magn Reson Med 2004;52:1388–1396. 15562493 10.1002/mrm.20288

[12] Skare S , Newbould RD , Clayton DB , Bammer R . Propeller EPI in the other direction. Magn Reson Med 2006;55:1298–1307. 16676335 10.1002/mrm.20890

[13] Atkinson D , Counsell S , Hajnal JV , Batchelor PG , Hill DLG , Larkman DJ . Nonlinear phase correction of navigated multi‐coil diffusion images. Magn Reson Med 2006;56:1135–1139. 16986111 10.1002/mrm.21046

[14] Lee C , Li Z , Pipe JG , Debbins JP . Turboprop+: enhanced Turboprop diffusion‐weighted imaging with a new phase correction. Magn Reson Med 2013;70:497–503. 23023533 10.1002/mrm.24481

[15] Robson MD , Anderson AW , Gore JC . Diffusion‐weighted multiple shot echo planar imaging of humans without navigation. Magn Reson Med 1997;38:82–88. 9211383 10.1002/mrm.1910380113

[16] Porter DA , Heidemann RM . High resolution diffusion‐weighted imaging using readout‐segmented echo‐planar imaging, parallel imaging and a two‐dimensional navigator‐based reacquisition. Magn Reson Med 2009;62:468–475. 19449372 10.1002/mrm.22024

[17] Holdsworth SJ , Skare S , Newbould RD , Bammer R . Robust GRAPPA‐accelerated diffusion‐weighted readout‐segmented (RS)‐EPI. Magn Reson Med 2009;62:1629–1640. 19859974 10.1002/mrm.22122 PMC2904969

[18] Oshio K . vGRASE: separating phase and T(2) modulations in 2D. Magn Reson Med 2000;44:383–386. 10975889 10.1002/1522-2594(200009)44:3<383::aid-mrm7>3.0.co;2-i

[19] Heidemann RM , Porter DA , Anwander A , Feiweier T , Heberlein K , Knosche TR , Turner R . Diffusion imaging in humans at 7T using readout‐segmented EPI and GRAPPA. Magn Reson Med 2010;64:9–14. 20577977 10.1002/mrm.22480

[20] Frost R , Porter DA , Miller KL , Jezzard P . Implementation and assessment of diffusion‐weighted partial Fourier readout‐segmented echo‐planar imaging. Magn Reson Med 2012;68:441–451. 22535706 10.1002/mrm.23242

[21] Larkman DJ , Hajnal JV , Herlihy AH , Coutts GA , Young IR , Ehnholm G . Use of multicoil arrays for separation of signal from multiple slices simultaneously excited. J Magn Reson Imaging 2001;13:313–317. 11169840 10.1002/1522-2586(200102)13:2<313::aid-jmri1045>3.0.co;2-w

[22] Feinberg DA , Setsompop K . Ultra‐fast MRI of the human brain with simultaneous multi‐slice imaging. J Magn Reson 2013;229:90–100. 23473893 10.1016/j.jmr.2013.02.002 PMC3793016

[23] Roemer PB , Edelstein WA , Hayes CE , Souza SP , Mueller OM . The NMR phased array. Magn Reson Med 1990;16:192–225. 2266841 10.1002/mrm.1910160203

[24] Pruessmann KP , Weiger M , Scheidegger MB , Boesiger P . SENSE: sensitivity encoding for fast MRI. Magn Reson Med 1999;42:952–962. 10542355

[25] Breuer FA , Blaimer M , Heidemann RM , Mueller MF , Griswold MA , Jakob PM . Controlled aliasing in parallel imaging results in higher acceleration (CAIPIRINHA) for multi‐slice imaging. Magn Reson Med 2005;53:684–691. 15723404 10.1002/mrm.20401

[26] Nunes RG , Hajnal JV , Golay X , Larkman DJ . Simultaneous slice excitation and reconstruction for single shot EPI. In Proceedings of the 14th Annual Meeting of ISMRM, Seattle, Washington, USA, 2006. Abstract 293.

[27] Setsompop K , Gagoski BA , Polimeni JR , Witzel T , Wedeen VJ , Wald LL . Blipped‐controlled aliasing in parallel imaging for simultaneous multislice echo planar imaging with reduced g‐factor penalty. Magn Reson Med 2012;67:1210–1224. 21858868 10.1002/mrm.23097 PMC3323676

[28] Moeller S , Yacoub E , Olman CA , Auerbach E , Strupp J , Harel N , Ugurbil K . Multiband multislice GE‐EPI at 7 Tesla, with 16‐fold acceleration using partial parallel imaging with application to high spatial and temporal whole‐brain fMRI. Magn Reson Med 2010;63:1144–1153. 20432285 10.1002/mrm.22361 PMC2906244

[29] Setsompop K , Cohen‐Adad JA , McNab JA , Gagoski B , Wedeen VJ , Wald LL . Improving SNR per unit time in diffusion Imaging using a blipped‐CAIPIRINHA simultaneous multi‐slice EPI acquisition. In Proceedings of the 18th Annual Meeting of ISMRM, Stockholm, Sweden, 2010. Abstract 187.

[30] Feinberg DA , Moeller S , Smith SM , Auerbach E , Ramanna S , Glasser MF , Miller KL , Ugurbil K , Yacoub E . Multiplexed echo planar imaging for sub‐second whole brain FMRI and fast diffusion imaging. PLoS One 2010;5:e15710. 21187930 10.1371/journal.pone.0015710 PMC3004955

[31] Setsompop K , Bilgic B , Cohen‐Adad J , Tisdall MD , Keil B , Witzel T , Rathi Y , Wedeen VJ , Adalsteinsson E , Wald LL . Whole‐brain DSI in 4 minutes: sparse sampling in q‐space with simultaneous multi‐slice acquisition. In Proceedings of the 20th Annual Meeting of ISMRM, Melbourne, Victoria, Australia, 2012. Abstract 693.

[32] Koopmans PJ , Boyacioglu R , Barth M , Norris DG . Whole brain, high resolution spin‐echo resting state fMRI using PINS multiplexing at 7T. Neuroimage 2012;62:1939–1946. 22683385 10.1016/j.neuroimage.2012.05.080

[33] Frost R , Porter DA , Douaud G , Jezzard P , Miller KL . Reduction of diffusion‐weighted readout‐segmented EPI scan time using a blipped‐CAIPI modification. In Proceedings of the 20th Annual Meeting of ISMRM, Melbourne, Australia, 2012. Abstract 116.

[34] Morelli JN , Runge VM , Feiweier T , Kirsch JE , Williams KW , Attenberger UI . Evaluation of a modified Stejskal‐Tanner diffusion encoding scheme, permitting a marked reduction in TE, in diffusion‐weighted imaging of stroke patients at 3 T. Invest Radiol 2010;45:29–35. 19996756 10.1097/RLI.0b013e3181c65c11

[35] Stejskal EO , Tanner JE . Spin diffusion measurements: spin echoes in the presence of a time‐dependent field gradient. J Chem Phys 1965;42:288–292.

[36] Nguyen Q , Clemence M , Ordidge RJ . The use of intelligent re‐acquisition to reduce scan time in MRI degraded by motion. In Proceedings of the 6th Annual Meeting of ISMRM, Sydney, Austrailia, 1998. Abstract 134.

[37] Porter DA . 2D‐navigator‐based re‐acquisition for motion artefact suppression in multi‐shot, diffusion‐weighted imaging. In Proceedings of the 14th Annual Meeting of ISMRM, Seattle, Washington, USA, 2006. Abstract 1047.

[38] Jones DK , Horsfield MA , Simmons A . Optimal strategies for measuring diffusion in anisotropic systems by magnetic resonance imaging. Magn Reson Med 1999;42:515–525. 10467296

[39] Jones DK . The effect of gradient sampling schemes on measures derived from diffusion tensor MRI: a Monte Carlo study. Magn Reson Med 2004;51:807–815. 15065255 10.1002/mrm.20033

[40] Behrens TEJ , Woolrich MW , Jenkinson M , Johansen‐Berg H , Nunes RG , Clare S , Matthews PM , Brady JM , Smith SM . Characterization and propagation of uncertainty in diffusion‐weighted MR imaging. Magn Reson Med 2003;50:1077–1088. 14587019 10.1002/mrm.10609

[41] Smith SM . Fast robust automated brain extraction. Hum Brain Mapp 2002;17:143–155. 12391568 10.1002/hbm.10062 PMC6871816

[42] Behrens TEJ , Berg HJ , Jbabdi S , Rushworth MFS , Woolrich MW . Probabilistic diffusion tractography with multiple fibre orientations: what can we gain? Neuroimage 2007;34:144–155. 17070705 10.1016/j.neuroimage.2006.09.018 PMC7116582

[43] Haacke EM , Brown RW , Thompson MR , Venkatesan R . Magnetic resonance imaging: physical Principles and Sequence Design. New York: Wiley‐Liss; 1999.

[44] Wansapura JP , Holland SK , Dunn RS , Ball WSJ . NMR relaxation times in the human brain at 3.0 tesla. J Magn Reson Imaging 1999;9:531–538. 10232510 10.1002/(sici)1522-2586(199904)9:4<531::aid-jmri4>3.0.co;2-l

[45] Peters AM , Brookes MJ , Hoogenraad FG , Gowland PA , Francis ST , Morris PG , Bowtell R . T2* measurements in human brain at 1.5, 3 and 7 T. Magn Reson Imaging 2007;25:748–753. 17459640 10.1016/j.mri.2007.02.014

[46] Cox EF , Gowland PA . Measuring T2 and T2' in the brain at 1.5T, 3T, and 7T using a hybrid gradient echo‐spin echo sequence. In Proceedings of the 16th Annual Meeting of ISMRM, Toronto, Canada, 2008. Abstract 1411.

[47] Andersson JLR , Skare S , Ashburner J . How to correct susceptibility distortions in spin‐echo echo‐planar images: application to diffusion tensor imaging. Neuroimage 2003;20:870–888. 14568458 10.1016/S1053-8119(03)00336-7

[48] Conolly S , Nishimura D , Macovski A , Glover G . Variable‐rate selective excitation. J Magn Reson (1969) 1988;78:440–458.

[49] Setsompop K , Cohen‐Adad J , Gagoski BA , Raij T , Yendiki A , Keil B , Wedeen VJ , Wald LL . Improving diffusion MRI using simultaneous multi‐slice echo planar imaging. Neuroimage 2012;63:569–580. 22732564 10.1016/j.neuroimage.2012.06.033 PMC3429710

[50] Norris DG , Koopmans PJ , Boyacioglu R , Barth M . Power Independent of Number of Slices (PINS) radiofrequency pulses for low‐power simultaneous multislice excitation. Magn Reson Med 2011;66:1234–1240. 22009706 10.1002/mrm.23152

[51] Eichner C , Setsompop K , Koopmans PJ , Lützkendorf R , Norris DG , Turner R , Wald LL , Heidemann RM . Slice accelerated diffusion‐weighted imaging at ultra‐high field strength. Magn Reson Med 2014;71:1518–1525. 23798017 10.1002/mrm.24809

[52] Poser BA , Anderson RJ , Guerin B , Setsompop K , Deng W , Mareyam A , Serano P , Wald LL , Stenger VA . Simultaneous multislice excitation by parallel transmission. Magn Reson Med 2014;71:1416–1427. 23716365 10.1002/mrm.24791 PMC3830622

[53] Wu X , Schmitter S , Auerbach EJ , Moeller S , Ugurbil K , Van de Moortele P . Simultaneous multislice multiband parallel radiofrequency excitation with independent slice‐specific transmit B1 homogenization. Magn Reson Med 2013;70:630–638. 23801410 10.1002/mrm.24828 PMC3884042

