# Supplementary material for: Scan time reduction for readout‐segmented EPI using simultaneous multislice acceleration: Diffusion‐weighted imaging at 3 and 7 Tesla
Source: Magn Reson Med. 2014 Jul 30;74(1):136–49. doi: 10.1002/mrm.25391 (PMC4854329; doi:10.1002/mrm.25391)
Supplement: Supplementary file 1 — Supplementary Information [file MRM-74-136-s001.doc]

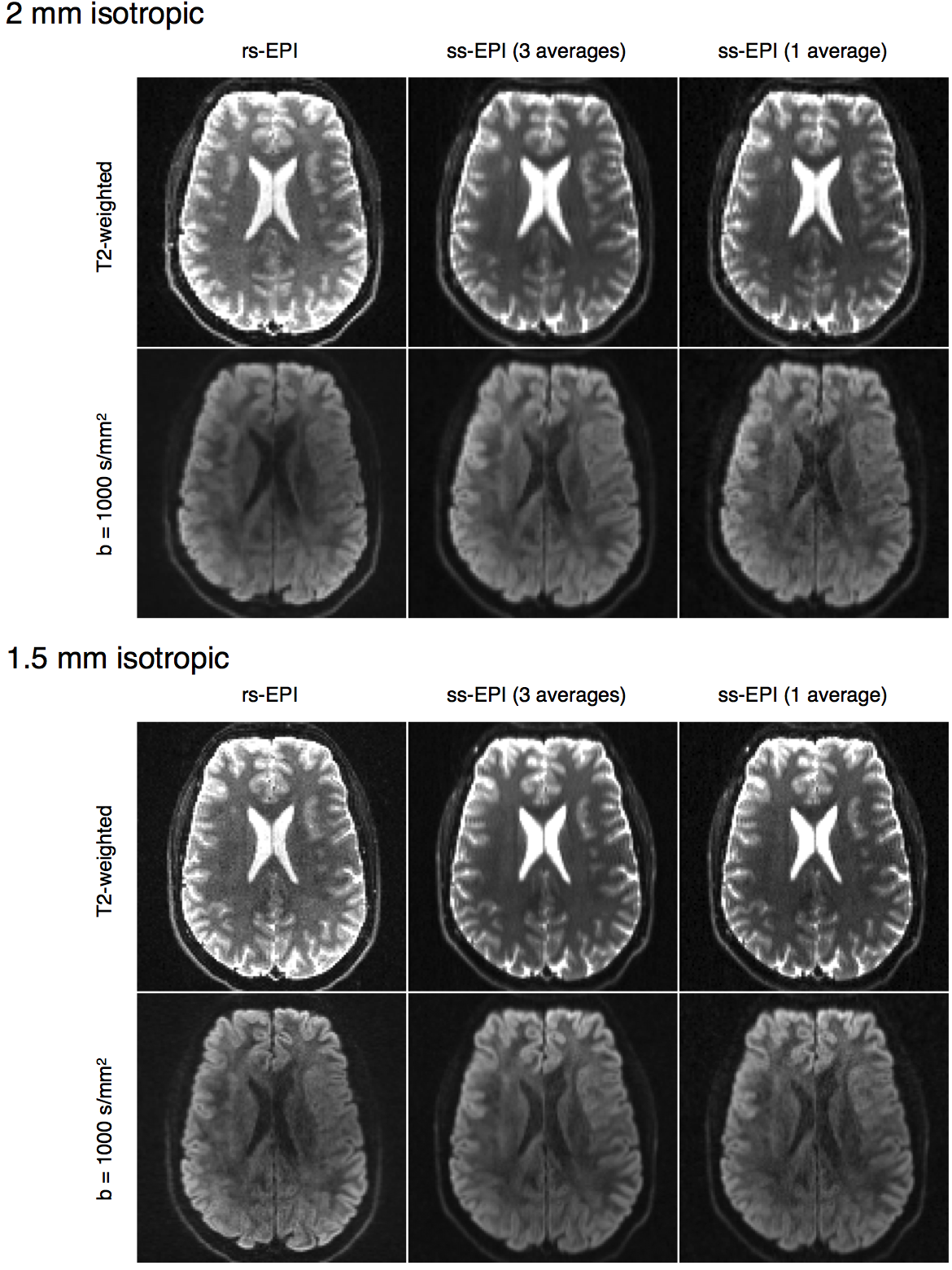


Supplementary Figure 1: Examples of raw *T_2_*-weighted and b = 1000 s/mm^2^ images acquired at 3 T with the ss-EPI and blipped-CAIPI rs-EPI DTI protocols with 2 mm and 1.5 mm isotropic resolutions.

Supplementary Figure 2:Comparison of blurring in the phase-encode (y) direction between SMS rs-EPI (left column) and ss-EPI (right column) with 1.5 mm isotropic resolution images acquired at 3 T and 1. 2 mm isotropic resolution images acquired at 7 T. Images of a resolution phantom are shown along with a plot of the signal profile at the position marked by the red dotted line.

Supplementary Figure 3:

Images demonstrating 3 T SMS rs-EPI with high in-plane resolution and thicker slices. Trace-weighted images of two healthy volunteers with (a) 0.9 and (b) 1.1 mm in-plane resolution. (c) 1.1×1.1×4 mm trace-weighted images showing a lesion in an 82-year-old patient who had suffered a TIA the previous day.

**Supplementary Table 1**

| Sequence | Nominal resolution | b=0 SNR | Estimated normalised pixel size | Theoretical voxel blurring | Theoretical effective PE resolution |
| --- | --- | --- | --- | --- | --- |
|  | (mm) |  |  | (%) | (mm) |
| 3 T DTI |  |  |  |  |  |
| rs-EPI | 2 | 13(8) | 1.00 | 17 | 2.34 |
| ss-EPI (1 av) | 2 | 14(8) | 1.27 | 24 | 2.48 |
| rs-EPI | 1.5 | 8(5) | 1.00 | 22 | 1.83 |
| ss-EPI (1 av) | 1.5 | 13(9) | 1.46 | 44 | 2.16 |
| 7 T DTI |  |  |  |  |  |
| rs-EPI | 1.2 | 11(10) | 1.00 | 41 | 1.69 |
| ss-EPI | 1.2 | 10(8) | 1.50 | 79 | 2.14 |

SNR and resolution analysis on the raw b=0 images from the DTI protocols. For each nominal resolution, the mean pixel size in the b=0 images was estimated and then normalised to the rs-EPI value. Theoretical % voxel blurring and the effective phase-encode resolution were calculated from the echo-train parameters in Table 1. The blurring in the ss-EPI images (affecting the normalised pixel size estimates) is a combination of T2* effects and smoothing due to the zero-filled phase-encode partial Fourier reconstruction.
